# Practice behavior of first-year university music students: thriving in unusual times

**DOI:** 10.3389/fpsyg.2026.1771837

**Published:** 2026-04-28

**Authors:** Manfred Nusseck, Frank Heuser, Claudia Spahn, Jutta Drinda, Adina Mornell

**Affiliations:** 1 Herb Alpert School of Music, University of California Los Angeles (UCLA), Los Angeles, CA, United States; 2 Instrumental and Vocal Music Education, University of Music and Theatre Munich, Munich, Germany; 3 Freiburg Institute for Musicians' Medicine, University of Music Freiburg, Medical Center and Faculty of Medicine – University of Freiburg, Freiburg Centre for Music Research and Teaching, Freiburg, Germany

**Keywords:** goal setting, music, pandemic, practice strategies, self-regulation

## Abstract

**Introduction:**

The way in which university music students practice is crucial for their development and critical for their lifelong success. Once they begin attending a music university, practice strategies change and evolve, influenced by individual and environmental factors. However, it remains unclear how music students' practicing changes during their first academic year and what might influence their practice behaviors.

**Method:**

In this study, music students completed questions about their practice both at the beginning and at the end of their first academic year at universities of music. Responses were collected in two surveys: in 2018–2019 and 2019–2020 in Germany (*n* = 63) and America (*n* = 47). A questionnaire was developed to allow participants to report their practice behaviors. It included items about daily practice time and the use of specific practice strategies and goals. The final measurement at the end of the second survey took place during the Covid-19 lockdown—quite an unusual situation for the students. Thus, the data also provide insights about how the special situation of the lockdown shaped participants' practice during the second semester of their first-year. Analyses considered comparisons between surveys and institutions.

**Results:**

During lockdown, all students increased their use of practice strategies related to self-regulated learning (SRL), considered the development of their own interpretations, and reduced the use of some less effective practice methods, such as repeating a piece from the beginning until a problem is solved. Furthermore, the results showed that music students surveyed in America reduced their average daily practice time at the end of their first academic year, while those in Germany increased it. This difference grew into a significant interaction effect between institutions.

**Discussion:**

The findings provide insights into music students' practice behaviors over their first academic year of higher education. It uncovered some variances in practice strategies that may have been a reaction to the challenges of the pandemic. Generally, much of the students' practice behaviors did not change. However, all appeared to become more self-regulated, even expanding their repertoire of practice methods in the lockdown term. Therefore, the findings suggest that students adapted their practice strategies when external circumstances changed in order to keep thriving during those unusual times.

## 1 Introduction

Musicians are aware that practice is crucial for the acquisition of musical skills and spend most of their musical time engaged in practicing. Studies showed that practice leads to the acquisition of musical skills and the attainment of expertise (Jørgensen and Hallam, 2016; Miksza, 2022). As a result, musicians adopt different approaches to practicing, which leads to a variety of outcomes (How et al., 2022).

Prior research on effective musical practice consistently indicated that quality is more important than quantity alone (McPherson et al., 2017). An analysis of videos made of pianists completing a retention test found that the strategies the musicians employ to learn their skills are more influential in determining future performance abilities than the pure amount of time spending practicing (Duke et al., 2009). However, questions remain on how musicians actually practice and how they acquire effective practice strategies.

In spite of the importance of developing productive practicing behaviors, Barry (2007) found in a qualitative study that even if music teachers demonstrated a variety of strategies during lessons, students employed only a limited number of them in their own practice sessions. According to their verbal descriptions of their practice, music students appeared to have a superficial understanding of their own learning strategies, which might be due to the fact they had received only little guidance on how to become effective learners. In order to know what and how to do the appropriate things at the correct moment, music students need to employ practice strategies that are multifaceted and individualized (Hatfield and Lemyre, 2016; McPherson et al., 2019; How et al., 2022). In contrast, practicing with ineffective strategies and unsuitable techniques such as trial and error can cost too much energy and lead to learning plateaus, motivational tiredness, poor habits, and even performance errors (Miksza, 2022).

Overall, there is a growing body of research that studies methods for efficient practice, e.g., to encourage self-regulated learning (SRL; Miksza et al., 2018; McPherson et al., 2019; Mornell et al., 2020; Osborne et al., 2021) or deliberate practice (Platz et al., 2014; Ericsson and Harwell, 2019). These studies focus on characteristics of specific practice strategies and describe why those practice approaches are more effective than others. However, they leave the question of how often musicians really use these strategies unanswered. In a descriptive study, Miksza and Brenner (2023) collected practice reports from advanced collegiate violinists who performed a self-learned new piece. The authors found that at the beginning of the learning the musicians seemed to struggle to implement SRL strategies. During the later stages of the practicing process, their practice strategies were more self-organized and more related to setting specific practice goals. When preparing for an orchestral audition, Kegelaers et al. (2022) found in a collective case study that the musicians were able to implement several different practice and performance strategies related to self-regulation approaches and increased their practice time.

### 1.1 Practice behaviors of university music students

Practice skills play a role not only in students' success in university music exams, but also in their future careers. Curricula at institutions of higher music education are tailored to the career goal of reaching professional musician status. Nevertheless, students spend a tremendous amount of their study time practicing and very little of that time is devoted specifically to the instruction of structure and strategy of practice (Jørgensen and Hallam, 2016).

The degree program at a music university can be very challenging. First-semester students inevitably will refine, change, and evolve their practice strategies, influenced by individual and environmental factors. Especially at the start of their studies, students are confronted with considerable challenges, including a new music teacher, a rather competitive social environment and an uncertain and challenging career (Gaunt, 2011). It has been shown that the first academic year is a physiologically and psychologically difficult phase for university music students (Spahn et al., 2017).

With regard to practice, few systematic long-term studies have examined changes between the start and the end of the first-year of study. In addition, it would be difficult to create and observe a control condition or one with different variables to compare changes of practice strategies within a regular first-year university curriculum with a similar but nevertheless different situation such as self-instruction or peer learning. The COVID-19 pandemic offered such a unique opportunity to investigate and compare strategies within the same cohort. While participants started their academic year as usual (Autumn 2019), the lockdown was proclaimed during the second semester (Spring 2020), and in-person lessons or social gatherings were prohibited in many countries. This created a disruption for musicians, especially with regard to maintaining their practice routines during that time (Hale et al., 2021).

Motivation and engagement in music learning at a music university depends largely on the learning environment (Evans and Bonneville-Roussy, 2016). During the pandemic, this environment changed dramatically from a more external and institutionally regulated environment to an internal and individually influenced situation. For instance, music teachers are often described as demanding, directive and controlling (Creech and Gaunt, 2012). During the lockdown, when in person tuition was not possible, students had a chance to adapt their individual learning and practice habits according to their own needs.

Much research on practice motivation is framed by the Self-Determination Theory (SDT; Evans and Ryan, 2022; Evans, 2023). Central to SDT, developed by Ryan and Deci (2000), is the belief that human motivation, personal growth, and pleasure rely on basic psychological needs (Vansteenkiste et al., 2020), guided by external and internal events. Motivation is considered on a continuum from controlled to autonomous motivation. Controlled settings with external and introjected regulations leads to a motivation or is seen to promote extrinsic motivation, whereas intrinsic regulation supports self-determined and autonomous motivation as well as enjoyment (Bonneville-Roussy and Evans, 2025). The latter can be achieved when some or all of three core psychological needs are satisfied (Evans and Ryan, 2022): autonomy (self-control on learning and in musical expression), competence (feeling effective and capable learning and performing), and relatedness (sense of belonging to a group and/or connected to others through music). Among university students, the satisfaction of basic psychological needs was found to be significantly associated with happiness (Vasiou et al., 2024) and impacts the quality of practice (Evans, 2023). For music students, it was found that control by the music teacher promotes extrinsically controlled practice motivation (externally regulated), while autonomous motivation was positively correlated with practice time and practice quality (Bonneville-Roussy and Evans, 2025). Thus, the question remains, whether a specific situation of unregulated practicing—as happened during the pandemic—endorsed forms of individual motivation, which enabled autonomous and productive—i.e., self-determined—practice in comparison to a regular situation.

Several studies have been conducted with musicians and music students with regard to the pandemic. Cohen and Ginsborg (2021), for example, found an increase in distress, confusion, and anxiety among UK musicians during the pandemic. Schiavio et al. (2021) concluded that Italian music students' practice strategies became more creative and collaborative during the pandemic, with technology facilitating communication between students who were practicing social distancing. Spiro and colleagues' study of adult UK music professionals (Spiro et al., 2021) described financial concerns as well as increased anxiety and loneliness. On the other hand, studies found that musicians' resilience increased with these difficult circumstances, resulting in positive adaptations and personal growth (Zhukov et al., 2024).

Other studies compared data collected before the pandemic with data measured during the pandemic. Most studies focused on changes in wellbeing, the satisfaction with Corona-measures instituted by the music university and the participation in study programs during the lockdown. For example, Rosset et al. (2021) reported an increase in stress in music students at a German university of music during the pandemic where their general mental health status was not different to before the pandemic.

Music-related studies mainly considered changes in the practice duration and motivation. In professional musicians, López-Íñiguez et al. (2022) found that self-motivated musicians were better able to manage their practice, whereas externally motivated musicians reduced practice duration during the lockdown. In young instrumental learners, the pandemic situation and remote learning reduced intrinsic motivation (Wieser and Müller, 2022). However, with a regression model they found that the age of the musicians was associated with external regulation: the older the learner, the lesser the extrinsic motivation.

With regard to music students, Rosset et al. (2021) found that 45% of them decreased their practice time during the pandemic. Some reasons for the decrease were a reduction in motivation and difficulties accessing a rehearsal room. In contrast, 31% of the music students actually increased their practice time, reporting that they used the free time during the lockdown for practice. A similar result was found by Fallowfield and Gomez (2022) with 41% of the Swiss music students reducing their practice time during the pandemic. Moreover, 55% of the students mainly intensified their technical practicing and report positive effects on their playing technique.

In another study, the mean practice duration of German music students was not different during the lockdown compared with before the lockdown (Nusseck and Spahn, 2021). While the average self-efficacy in music learning also remained unchanged, in self-regulated music learning, the degree of self-reflection on one's own learning progress increased during the lockdown. After the pandemic, music students steadily increased their practice time and replied to a comparative question that they had practiced less during lockdown but had since returned to their normal level (Spahn et al., 2022).

Although these studies reported differences between before and during the pandemic in university music students, it remains unclear whether these changes were part of a regular pattern, that is, due to the university curriculum, or whether their practice behavior had been influenced by the pandemic. The lockdown created a very special situation in which music students had no in person lessons and were mainly left to their own devices. Any comparison of practice between with and without a pandemic should also consider those changes in practice behaviors that ordinarily evolve over the course of a regular (non-pandemic) academic year.

### 1.2 Aim of the study

Knowing that university music students' practice is crucial for their development and lifelong success, the original interest of this study was to investigate how music students' practice behavior changes over the course of the first academic year. For that, a specific questionnaire on practice strategies and goals was developed. A further goal of the original exploratory study was to test this new questionnaire. As part of this first longitudinal study, music students were surveyed twice in the academic year 2018–2019, once at the beginning of their studies and a second time at the end of the second semester.

This original study was repeated in the following year to both increase sample size and apply analysis methods of reliability and validity. Thus, the survey was repeated in the subsequent term, i.e., the next academic year. By chance, the end point of this second-year coincided with the lockdown of the COVID pandemic. With this data, a comparison between a regular academic year (2018–2019) and a year with special circumstances (2019–2020) was performed.

The goal was to collect data on the *quality* of practice, i.e., how often music students use specific practice approaches and how frequently those specific strategies change during the first-year of study, with a look at potential effects of the *quantity* of practice as well. For that, the authors developed a questionnaire to evaluate daily practice time, certain practice strategies employed, sources consulted for practice methods, and goals of the practice. This questionnaire was used to assess the amount of SRL and deliberate practice methods as well as participants' opinions about the effectiveness of those methods.

Another interest of this study was whether music students with divergent curricula and study objectives may have changed their practice behavior, increasing their use of strategies related to self-regulation, during the pandemic. To this end, data from three institutions of higher music education, two German music universities (in Freiburg and Munich) and an American music university (UCLA), were used. The German music universities offer a predominantly traditional music education with a historical foundation in the areas of classical music, opera, and musicology. The main goal of music education for those music students studying the Bachelor of Music in Germany is to provide them with the necessary training to become professional orchestra musicians and/or music teachers. The students arrive with extensive individual musical training on an instrument or in voice and continue their education in one-on-one lessons and orchestras.

Although the American students participating in this study must demonstrate excellent performance skills to enter the School of Music, they must also have distinguished themselves academically in order to be accepted to UCLA, as opposed to other institutions and conservatories. Similar to their German counterparts, their study program includes one-on-one lessons, ensemble performance, music theory, and musicology. However, extensive courses in the humanities and sciences are also required to earn a UCLA bachelor's degree. Even though music making is a central component of these students' lives, achieving a full-time position in an orchestra may not be one of their goals. Many of them maintain other academic interests, with some choosing to simultaneously earn a second degree in an additional discipline.

These two sets of music students are of particular interest because all participants have chosen to study music at a university with the aim of obtaining a university degree in music, albeit with rather different approaches.

The main research questions posed in this study were:

- How does the practice behavior of music students change over their first academic year at a university of music?
- Could changes in practice strategies occurring during the special situation of the pandemic be associated with self-regulation?
- Which practice approaches changed between the regular and the special year?
- Are there practice behavioral differences between music students at the American and German music universities studied?

In other words, the objective of this study evolved over time, allowing a first glimpse at how an unusual situation such as the pandemic could affect music students' practice behavior and allowed a discussion about what these results might also tell us about practice behaviors during a regular year. Therefore, the study is not primarily focused on the *effects* of the pandemic on practice, but uses the unique situation of the lockdown—during which the students were forced to work outside of regulated environments—to compare and contrast changes in practice behaviors during a regular first academic year with those reported when the usual educational framework was disrupted. It was expected that in a situation with less controlled regulation, autonomous motivation would increase and support behaviors related to SRL.

## 2 Materials and methods

### 2.1 Procedure

Music students at the University of Music and Theatre Munich (HMTM), the University of Music Freiburg (HfMFr) and the Herb Alpert School of Music at the University of California Los Angeles (UCLA) were invited to participate in the study. The study was approved by the UCLA Institutional Review Board (IRB) and the Ethics Commission of the University Clinic Freiburg. Both certificates were provided to the HMTM administration, which then gave permission for the study on the basis of those approvals.

**Figure 1:**
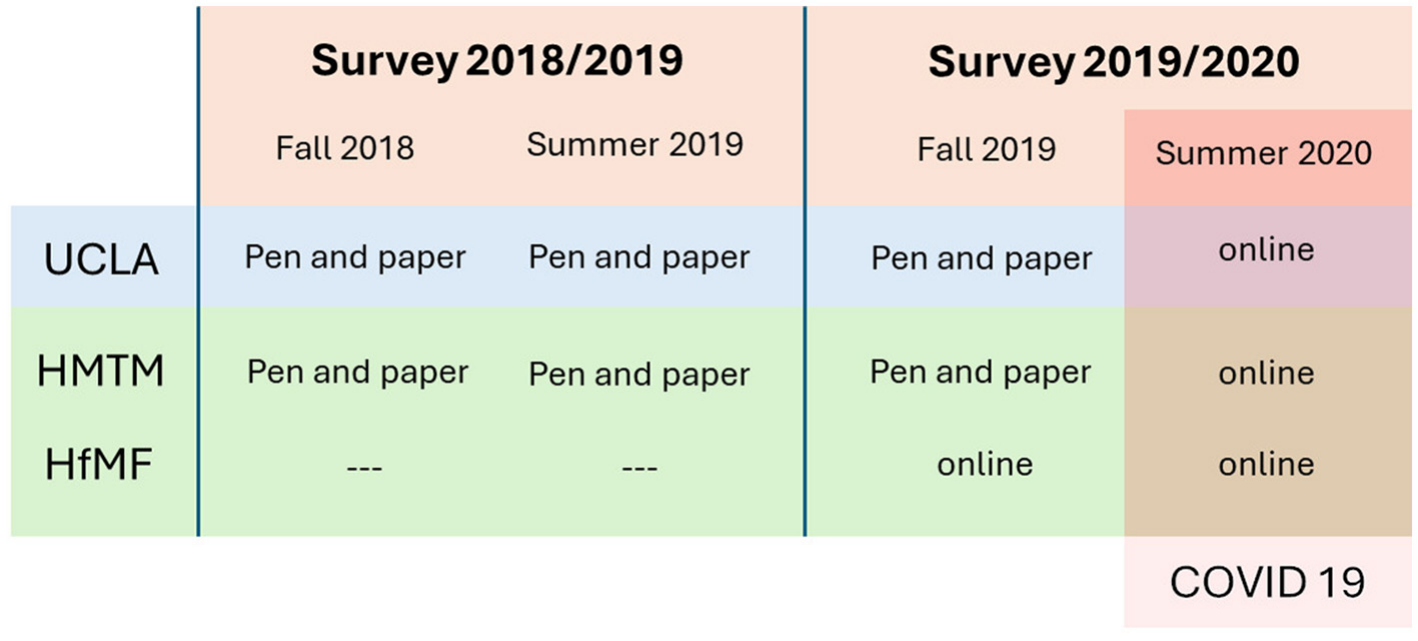
Schematic diagram of the survey and measurement design (UCLA, University of California LA; HMTM, University of Music Munich; HfMF, University of Music Freiburg).

A schematic overview of the surveys in this study is shown in Figure 1. The first survey was conducted starting in Fall 2018 and ending in Summer 2019. The second survey started in Fall 2019 and ended in Summer 2020. Both surveys included two measurements in which students completed a questionnaire, one at the beginning and one at the end of the respective academic year.

During the pandemic, strict lockdown rules were in place, beginning in March and ending in July or August 2020. Despite different regulations among the universities, the pandemic situation led to similar challenges for all music students, i.e., temporary closing of the university, no in-person lessons or rehearsals, and reduced social contacts.

The surveys in Fall 2018 and Summer 2019 were conducted using paper-and-pencil questionnaires handed out during classes with first-year students at the HMTM and UCLA. In Fall 2019, a paper-and-pencil questionnaire was again used at the HMTM and UCLA and at the HfMF, the first measurement was performed online, sending an email to students identified as studying in their first-year. In Summer 2020, participants at all institutions answered their questions online. The systems used for online questionnaires for the HMTM and UCLA were posted in both English and German using https://www.q-set.de and the same questions were used via https://www.soscisurvey.de at the HfMFr.

To connect the questionnaires in both measurements while retaining anonymity, participants were asked in the first measurement to create an individual code that was easy to remember and did not allow any identification about the person. This code was then requested in the second measurement and provided the possibility to link the questionnaires without access to personal information of the individuals.

### 2.2 Participants

For the analysis, participants from both German universities, i.e., HMTM and HfMFr, were combined into one data set and were evaluated as a single institution called “UMGer” (“Universities of Music Germany”). Participants included had started their Bachelor of Music in general music programs at the time of the first measurement. Students with theoretical study programs, such as music theory, music history or musicology, as well as composition and jazz students did not participate.

In the first survey (2018/2019), 79 students participated in the first measurement and 64 in the second measurement. All 64 participants in second measurement could be linked to the first measurement. In the second survey (2019/2020), 140 students participated in the first measurement and 63 in the second measurement. Only 46 participants could be linked to the first measurement (Table 1).

**Table 1:** Sample characteristics in both surveys.

Sample characteristics	Survey 2018/2019		Survey 2019/2020	
	UMGer	UCLA	UMGer	UCLA
Number of participants in the first measurement	43	36	78	62
Number of participants in the second measurement	34	30	47	16
Participants linked in both measurements	34	30	30	16
Mean age (years) with SD	20.1 (2.2)	18.5 (1.9)	20.4 (2.7)	18.0 (1.3)
Gender	F 62%	F 50%	F 63%	F 56%
	M 38%	M 50%	M 37%	M 44%

SD, standard deviation; F, female; M, male; UMGer, Universities of Music Germany; UCLA, University of California LA.

The mean age of the students at the German universities were significantly higher than the students at the UCLA in both surveys. However, there was no age difference at both universities between the surveys. At the German university, there were more female students than at UCLA, but there was also no distribution difference between the surveys.

### 2.3 Questionnaire

The self-developed questionnaire distributed focused on the practice behavior of music students. An introductory set of questions included general sociodemographic questions, i.e., age and gender, and music related questions, e.g., their main instrument.

In the first question, the participants were asked to rate their average daily practice duration in minutes. Then, they were asked if they employed specific practice strategies in their practice, given a list of seven options with a free-text field for individual methods not listed as the eights option. As a multiple-choice block, each item could be marked or left unmarked. The block was introduced with the sentence: “During my practice, I use the following strategies:” Three of the strategies presented considered a less effective practical strategy: “Play/sing through pieces without stopping,” “Repeat the piece until the problem is solved,” and “Stop when there is a problem and start over.” They can be described as “mindless practice strategies” according to Passarotto et al. (2022) describing purposeless repetitive behaviors. The next item was a task segmentation strategy (“Isolate and work on a problem”) with a clear goal to work on a particular problem. The last three items considered a modifying strategy with either varying the tempo (“Play/sing the piece slowly and gradually bring it up to tempo”), the key (“Transpose the piece or sections of the piece) and the rhythm (“Deliberately modify the printed rhythm of a section”).

In another block, there were questions regarding the origins of practice methods. The block provided a list of six potential sources and an option for other sources with a free text field introduced by: “When practicing, I use methods that I learned from:” The idea behind this block was to investigate whether the sources change during the first-year of study and where these sources shift to. This is particularly interesting when sources were lacking during the pandemic. The provided sources were the current teacher, a former teacher, master classes, ensemble directors/coaches/conductors, peers, and YouTube tutorials/videos. Similar to the previous block, the items were either marked if they applied or left unmarked.

In the third and last block, participants considered the main goals of practice. Here, general principles of practice were addressed. The items were introduced by: “I spent my practice time in the following manner:” The goals were “Working on general technical aspects,” “Playing/singing through the repertoire,” “Experimenting with and developing interpretation,” “Improving intonation,” and “Performing live for friends or taping my playing/singing.” Again, items could be marked or left unmarked.

### 2.4 Analysis

For the analyses, SPSS 30 (Armonk, NY: IBM Corp.) was used. Descriptive statistics were calculated for the practice time and the mean value with standard deviation (SD) are reported. The reported practice times followed a normal distribution according to the Kolmogorov–Smirnov test. For the practice items, the percentage of the agreement was calculated.

To analyze the changes in the practice aspects between the two measurements, repeated measure analyses (ANOVA) were performed. To investigate effects within the two surveys (Survey 1: 2018–2019 and Survey 2: 2019–2020), both surveys were analyzed separately with the repeated factor time (the two measurements) and the random factor institution (UMGer and UCLA). To additionally investigate different changes between the surveys, the institutions were analyzed separately with the repeated factor time and the random factor survey. Interactions within the factors were also calculated. The level of significance was set to *p* = 0.05. In addition to the *p*-value, the effect sizes are reported using the partial Eta^2^ value with ranges of < 0.05 for none to small effects, >0.05 for medium effects and >0.14 for large effects (Richardson, 2011).

## 3 Results

Since the questionnaire items were dichotomous, a tetrachoric correlation matrix was created and used to perform the Kaiser–Meyer–Olkin (KMO) test to determine whether the data were suitable for exploratory factor analysis (cut-off: Measure of Sampling Adequacy MSA > 0.7). Only the responses of the first measurements were used for this analysis. For the seven items addressing the practice strategies, the MSA was 0.38, for the six items practice methods, the MSA was 0.55, and for the five items on the goals of practicing, the MSA was 0.54. Therefore, performing a factor analysis with this dataset was not possible. For this study, item-level analyses were performed to allow for a descriptive account of changes in specific practice behaviors. With that, it was not possible to draw direct conclusions about the development of specific theoretical constructs.

The mean values of the practice time and the percentages of music students who performed each practice approach at the beginning and at the end of the first academic year by institution and survey are listed in Table 2. The statistical analyses separated by survey and by institution are shown in Tables 3, 4, respectively. In these, Tables only the significant effects were included. The complete tables can be found in the Supplementary material. The effect sizes of the significant effects were found to be medium to large.

**Table 2:** Mean values of the practice time with standard deviation and the percentages of agreed practice approach by survey, measurement, and institution.

Items of the questionnaire	Survey (2018/2019)				Survey (2019/2020)			
	UMGer (*n* = 34)		UCLA (*n* = 30)		UMGer (*n* = 30)		UCLA (*n* = 16)	
	*T1*	*T2*	*T1*	*T2*	*T1*	*T2*	*T1*	*T2*
Daily practice time (minutes)	134.3 (56.8)	143.2 (58.0)	105.0 (39.8)	97.5 (53.3)	147.0 (71.4)	161.7 (68.8)	104.7 (52.4)	80.6 (28.4)
During my practice, I use the following strategies:								
Play/sing through without break	65%	74%	90%	93%	67%	80%	75%	100%
Repeat until problem is solved	15%	12%	37%	23%	33%	13%	75%	63%
Stop when there is a problem and start over	18%	15%	23%	33%	57%	30%	87%	87%
Identify problem and work on it	97%	88%	94%	97%	93%	100%	100%	100%
Start slowly and increase tempo	97%	94%	100%	97%	100%	97%	87%	100%
Transpose the piece or sections of the piece	6%	12%	16%	13%	23%	33%	20%	7%
Modify the rhythm of the piece or a section	59%	74%	68%	74%	73%	77%	75%	63%
When practicing, I use methods that I learned from:								
Current teacher	91%	91%	81%	84%	100%	97%	93%	97%
Former teacher	85%	82%	61%	77%	97%	97%	94%	81%
Master classes	35%	38%	42%	52%	46%	43%	63%	63%
Ensemble conductor	24%	32%	32%	29%	24%	48%	50%	50%
Peers	35%	59%	55%	65%	24%	41%	75%	56%
Video tutorials	0%	12%	23%	23%	24%	21%	50%	56%
I spent my practice time in the following manner:								
General technique	94%	91%	90%	94%	93%	97%	93%	100%
Playing/sing through repertoire	62%	65%	65%	65%	52%	48%	56%	44%
Experimental interpretations	68%	74%	65%	65%	66%	79%	63%	81%
Improving intonation	56%	59%	63%	60%	66%	66%	75%	75%
Performing (for friends or recording)	53%	53%	40%	43%	48%	52%	50%	69%

UMGer, Universities of Music Germany; UCLA, University of California LA; T1/T2, time of measurement at the beginning and the end of the academic year, respectively.

**Table 3:** Statistical parameters (*p*-values and effect sizes) of the main effects and the interaction effects for each survey by measurement and institution.

Statistical analyses (*p*-values and p.eta^2^ in italic)	Survey (2018–2019)			Survey (2019–2020)		
	Time (*T*1–*T*2)	Institute	Inter-action	Time (*T*1–*T*2)	Institute	Inter-action
Daily practice time	n.s.	**0.004**	n.s.	n.s.	**0.001**	**0.012**
		*0.128*			*0.216*	*0.136*
During my practice, I use the following strategies:						
Play/sing through without break	n.s.	**0.007**	n.s.	**0.025**	n.s.	n.s.
		*0.110*		*0.111*		
Repeat until problem is solved	n.s.	n.s.	n.s.	**0.022**	**< 0.001**	n.s.
				*0.112*	*0.255*	
Stop when there is a problem and start over	n.s.	n.s.	n.s.	n.s.	**< 0.001**	n.s.
					*0.266*	
Identify problem and work on it	n.s.	n.s.	n.s.	n.s.	n.s.	n.s.
Start slowly and Increase tempo	n.s.	n.s.	n.s.	n.s.	n.s.	n.s.
Transpose the piece or sections of the piece	n.s.	n.s.	n.s.	n.s.	n.s.	n.s.
Modify the rhythm of the piece or a section	n.s.	n.s.	n.s.	n.s.	n.s.	n.s.
When practicing, I use methods that I learned from:						
Current teacher	n.s.	n.s.	n.s.	n.s.	n.s.	n.s.
Former teacher	n.s.	n.s.	n.s.	n.s.	n.s.	n.s.
Master classes	n.s.	n.s.	n.s.	n.s.	n.s.	n.s.
Ensemble conductor	n.s.	n.s.	n.s.	n.s.	n.s.	n.s.
Peers	**0.017**	n.s.	n.s.	n.s.	**0.007**	n.s.
	*0.087*				*0.157*	
Video tutorials	n.s.	**0.037**	n.s.	n.s.	**0.023**	n.s.
		*0.067*			*0.114*	
I spent my practice time in the following manner:						
General technique	n.s.	n.s.	n.s.	n.s.	n.s.	n.s.
Playing/sing through repertoire	n.s.	n.s.	n.s.	n.s.	n.s.	n.s.
Experimental interpretations	n.s.	n.s.	n.s.	**0.035**	n.s.	n.s.
				*0.099*		
Improving intonation	n.s.	n.s.	n.s.	n.s.	n.s.	n.s.
Performing (for friends or recording)	n.s.	n.s.	n.s.	n.s.	n.s.	n.s.

n.s., not significant; p.eta^2^, partial Eta square; all effect sizes of n.s. were < 0.05; T1/T2, time of measurement at the beginning and the end of the academic year, respectively. Bold values indicate significant effects with *p* < 0.05.

**Table 4:** Statistical parameters (*p*-values and effect sizes) of the main effects and the interaction effects for each institution by measurement and survey.

Statistical analyses (*p*-values and p.eta^2^ in italic)	UMGer			UCLA		
	Time (*T*1–*T*2)	Survey (*M*1–*M*2)	Inter-action	Time (*M*1–*M*2)	Survey (*M*1–*M*2)	Inter-action
Daily practice time	**0.029**	n.s.	n.s.	**0.022**	n.s.	n.s.
	*0.074*			*0.119*		
During my practice, I use the following strategies:						
Play/sing through without break	n.s.	n.s.	n.s.	**0.004**	n.s.	**0.024**
				*0.174*		*0.110*
Repeat until problem is solved	**0.012**	n.s.	n.s.	n.s.	**0.003**	n.s.
	*0.097*				*0.189*	
Stop when there is a problem and start over	**0.019**	**0.003**	n.s.	n.s.	**< 0.001**	n.s.
	*0.086*	*0.132*			*0.395*	
Identify problem and work on it	n.s.	**0.029**	n.s.	n.s.	n.s.	n.s.
		*0.075*				
Start slowly and Increase tempo	n.s.	n.s.	n.s.	n.s.	n.s.	n.s.
Transpose the piece or sections of the piece	n.s.	**0.012**	n.s.	**0.039**	n.s.	n.s.
		*0.098*		*0.093*		
Modify the rhythm of the piece or a section	n.s.	n.s.	n.s.	n.s.	n.s.	n.s.
When practicing, I use methods that I learned from:						
Former teacher	n.s.	n.s.	n.s.	n.s.	n.s.	n.s.
Current teacher	n.s.	n.s.	n.s.	n.s.	n.s.	n.s.
Master classes	n.s.	n.s.	n.s.	n.s.	n.s.	n.s.
Ensemble conductor	**0.014**	n.s.	n.s.	n.s.	n.s.	n.s.
	*0.096*					
Peers	**0.007**	n.s.	n.s.	n.s.	n.s.	n.s.
	*0.114*					
Video tutorials	n.s.	**0.028**	n.s.	n.s.	**0.029**	n.s.
		*0.076*			*0.102*	
I spent my practice time in the following manner:						
General technique	n.s.	n.s.	n.s.	n.s.	n.s.	n.s.
Playing/sing through repertoire	n.s.	n.s.	n.s.	n.s.	n.s.	n.s.
Experimental interpretations	n.s.	n.s.	n.s.	n.s.	n.s.	n.s.
Improving intonation	n.s.	n.s.	n.s.	n.s.	n.s.	n.s.
Performing (for friends or recording)	n.s.	n.s.	n.s.	n.s.	n.s.	n.s.

n.s., not significant; p.eta^2^, partial Eta square; all effect sizes of n.s. were < 0.05; UMGer, Universities of Music Germany; UCLA, University of California LA; T1/T2, time of measurement at the beginning and the end of the academic year, respectively; S1, survey 2018–2019; S2: survey 2019–2020. Bold values indicate significant effects with *p* < 0.05.

The practice time increased significantly between the start and the end of the academic year for the students at the UMGer in both surveys without significant interaction. For the students at the UCLA, the mean practice time decreased to the end of the academic year in both surveys, also without significant interaction. The different changes in practice time at the two institutions revealed a significant interaction effect in the second survey.

In the practice strategies, the two most performed strategies were the task segmentation strategy of identifying a problem and working on it and the manipulation strategy of steadily increasing the tempo. This was found across all music students and independent of time and lockdown situation.

Running through a piece without a break was performed more often by the students at the UCLA. In the pandemic year, they even significantly increased the use of this strategy toward the end of the academic year in comparison to the students at the UMGer.

The strategy of repeating the piece until the problem is solved was used less frequently at the end of the academic year by all music students. It even significantly reduced in the second survey, but there was no significant difference in the changes across the academic year between the surveys.

The most consulted source for practice methods was the current teacher followed by the former teacher. Despite the pandemic situation, both sources were used extensively without significant differences. The number of methods learned from fellow students increased at the end of the academic year in the first survey. In the pandemic year, the number did not increase much and even decreased for the music students at the UCLA. Video tutorials were used the least frequently and differed significantly in number between institutions.

With respect to practice goals, music students mostly focused on technique. There were no significant differences in the practice goals between the measurement points of the academic year and the surveys. The only exception was for the experimenting with and developing of interpretations with a significant increase toward the end of the academic year with the pandemic.

## 4 Discussion

This study investigated changes in practice behavior within the first academic year of a small sample of entry level university music students. Preparation methods, typical problem-solving strategies, and evolving learning behaviors were assessed using a questionnaire in a longitudinal design. Questions were designed to consider specific approaches to practice and behavioral strategies that students might employ in the first-year of study. When the COVID-19 pandemic occurred in 2020, the replication study was also used to investigate whether the special circumstance of the lockdown yielded different practice behaviors. The results offer a picture of how music students' practice in a regular academic year might evolve, and how practice changed during the pandemic, when students were forced to quarantine, disrupting their daily practice routines and limiting or blocking their access to instruction.

With regard to the amount of time spent practicing during the pandemic, other studies found contradicting results. Increases in practice time were interpreted as using the free time to practice more and decreases were explained by reduced motivation (Rosset et al., 2021; Fallowfield and Gomez, 2022). However, these were interpretations based on a single measurement point of observed behavior during lockdown. In contrast, this study was conducted both before and during the pandemic. The results reported here show no significant difference in the change of practice time during the pandemic in comparison to the situation without the pandemic. The practice time itself can therefore not be used to sufficiently explain strategies and motivation in practice behavior in unusual times.

The changes in the mean daily practice time differed between the music students at both institutions. While music students at the UMGer increased their practice time, it decreased among students at the UCLA, albeit in the same way in both academic years with and without pandemic. Only for the music students at the UCLA the practice time reduced slightly more. This finding suggests that the special circumstances of the pandemic did not have a general impact on music students' practice time in terms of their usual practice habit.

As to the questions of practice strategies employed by the music students, the most frequently used strategies were to identify a problem with specifically working on it and to slowly increase the tempo. Both strategies are considered as effective strategies (Passarotto et al., 2022) and were executed similarly in both academic years with and without pandemic. This indicates that these practice approaches had been internalized by the students at both institutions and can also be applied in unusual situations such as the pandemic.

The more ineffective, more mindless practice strategy according to Passarotto et al. (2022) of playing through a piece without a break was often employed by the music students. The use of this strategy increased by the end of the academic year in both surveys. In the year with the pandemic, the students at the UCLA increased this strategy significantly in comparison to the students at the UMGer. Playing a piece all the way through can be used to simulate performance situations, but use of this strategy alone may result in less effective learning. Playthroughs should be used to detect problematic sections and to work on them separately. However, it seems that this strategy gained even more popularity during the pandemic, possibly due to reduced number of lessons and performances or probably just as a way to pass time. This may have led to fewer opportunities for feedback, thus leaving students alone with their self-reflection, causing them to “default” to playthroughs.

Two additional and less effective practice strategies were: (a) repeating the piece until the problem disappears and (b) stopping when a problem occurs and starting over. These were used less frequently at the end of both academic years. During the pandemic, participants reduced the implementation of these strategies even further. It seems that music students learned within the first semester that these are not useful practice strategies, and even without direction from a teacher during the pandemic, they did not adapt or customize them.

Regarding the sources for practice behavior, music students mainly relied on the methods taught by their teachers, both current and former. In the academic year before the pandemic, the music students increased the use of methods they observed being used by other music students. This suggests that the influence of fellow students at the university of music should not be underestimated. Despite the clear primary source being the instrumental teachers, peers play also an important role. This is particularly evident for the students at the UCLA. In the academic year affected by the pandemic, when in person contact with fellow students was lacking, the students at the UCLA showed a reduced use of this resource at the end of the year. In contrast, the students at the UMGer were still able to increase their contact with peers and use them as a source of practice ideas.

Coincidence or not, in the second academic year surveyed, video tutorials were used more often as a source for learning in comparison to the first survey. Within both surveys, there was no difference between the measurements (between the beginning and the end of semester). It might have been expected that the use of videos would increase during the pandemic, when musicians were more or less in isolation. However, the results showed that the use of videos did not increase.

When asked about their practice goals, music students reported focusing mainly on *general* technical aspects. This and most of the other goals did not differ between the academic years with and without pandemic at both institutions. The goals stayed the same, except for the manner of experimentation with and development of interpretation. The use of this goal increased significantly in the pandemic year. When students had to practice on their own, they seemed to have evolved a sense for self-evaluation and self-judgement of their musical interpretation. This result suggests that in a normal year, interpretative decisions were mainly driven by the teacher. Without regular lessons, the students seemed to be more engaged in the creative process, developing their own interpretations of the music.

Hatfield (2016) showed that bachelor music students had little to no experience with planning and goal setting for their practice, but with an intervention containing SRL approaches, participants' concentration, self-observation and self-efficacy increased. A similar result was found with piano students (Pike, 2017). Those studies show that it is important to include instruction in learning strategies in the main bachelor curriculum at music universities. In the study conducted by Miksza and Brenner (2023), music students found it difficult to implement self-regulation learning approaches. However, they seemed to “know” about effective self-regulation approaches but were less able to “do” them at first. Once they understood more about the effectiveness of SRL, they started to implement those methods. Miksza and Brenner suggest that the external regulations of the teachers had weakened the students' ability to engage in self-determined practice. When left alone, they developed more effective practice strategies. This appears to be confirmed by the findings of this study. While Kegelaers et al. 92022) found, that if it is important to them (e.g., an orchestral audition), musicians were able to employ effective self-regulation methods. It suggests that teachers may need to nurture a change of mindset so that music students include SRL approaches from the start in their daily practice.

The findings in this study indicate a shift toward more self-directed practice during the pandemic. This might be due to the specific situation in which music students had more autonomy and freedom in their practice (c.f., Evans, 2023). It is also possible, that this independence was just an adaptive response to a situation requiring increased personal organization and responsibility. Regarding the other psychological needs considered in SDT, relatedness, i.e., the connectedness with others, can barely be considered during the pandemic, as contact with others was reduced. While other studies showed that the lack of external regulation reduced practice motivation (Wieser and Müller, 2022), this study reported that the music students increased their use of practice methods related to intrinsic motivation. However, neither the questions posed nor the number of participants allow more than speculation.

No substantial differences were found between the UMGer and UCLA university systems, with respect to first semester learning cultures as well as time and quality of practice. Interestingly, with less external input, i.e., when they were left to work primarily their own, some students' behaviors changed in a positive way: They increased their use of SRL strategies. Yet, participants' practice behavior was also negatively modified, as there was a slight increase of less effective strategies, such as playing through a piece from start to finish. With respect to daily practice time, a reduction is often interpreted as a sign of ineffectiveness, of not wanting to improve one's skills. However, it has been shown that the quantity of practice time alone is less important (McPherson et al., 2017) than the quality. This study supports those findings. Students at the UMGer practiced longer and students at the UCLA practiced fewer hours. Yet, despite differences in daily practice time, both groups showed an increase in the number of strategies employed and improvement in the range of goals described.

### 4.1 Limitations

The biggest limitation of this study is the small sample size. Even though it is generally not easy to recruit music students for long-term studies, it was even more difficult to find and keep participants during the difficult time of the pandemic. At UCLA, the number of music student participating represents about a third of the first semester students. Thus, these findings do not claim to be representative for larger groups or other institutions. They are relevant only to the described sample and limited in analysis, interpretation, and applicability to other groups.

In addition, there was a high risk of alpha error (Type II error) due to the number of analyses performed. A suitable analysis method could have been a linear mixed model, but this was not performed due to the small sample sizes. To emphasize the significant effects, the effect sizes were reported and, as they were all of a medium-to-large level, the interpretation was based on these significant effects. Furthermore, due to the unique circumstances surrounding the pandemic, the study can, hopefully, not be replicated in order to increase the sample size.

The questionnaire used was rather rudimental. Due to the small sample sizes in all surveys, explorative factor analyses were not possible to perform. Therefore, no reliability or validity was analyzed. The use of a Likert-scale would have improved the questionnaire and provided more detailed information of the item difficulties. Due to the missing factor analysis, the results were based on individual item analyses which limited the interpretations to changes in individual practice behaviors rather than validated coherent psychometrically constructs. For future studies, the questionnaire needs to be validated with a larger sample to draw connections to other questionnaires regarding deliberate practice, SRL, and SDT. A revision of the questionnaire that both addresses these limitations and includes standardized questionnaires is recommended for future studies.

### 4.2 Conclusion

The pandemic was a unique and potentially traumatic experience for music students. Surveys such as the one presented here, however, offer opportunities to examine potential differences in learning behaviors. The interpretations of the results of this study should be viewed with caution due to the small sample size. However, they may offer useful insights into university music students' practice behaviors when one considers how students reacted to the challenges of the pandemic.

Regarding theories of SRL (McPherson et al., 2019), the results show that the music students do indeed have an understanding of effective strategies and implement them, even in unusual situations when directives from their teachers were unavailable and they need to practice on their own. Most of the students did not significantly change their practice behaviors during the pandemic. Most of the practice approaches reported were similar across both academic years—with and without pandemic. However, some participants' strategies improved, with goals refined, during the pandemic. This appears to indicate that with less influence of teachers, that is, with less external feedback, music students choose to increase their use of SRL and practice techniques and find their own motivation.

Based on the results reported here, the authors recommend that music educators expand their teaching to include the presentation of—and arguments for—self-regulated practice methods. Support for SRL should be included in the goals of the university music education system. Music students should be encouraged to focus on a variety of self-driven practice strategies from the very beginning of their university training. When students know both how to practice and how to sustain their motivation—even without continuous support of their teachers—they will not only use their time more effectively, but they will also be more creative, better motivated and able to flourish. In short, they will be able to thrive in any situation that comes their way, whether planned or unexpected.

## Data Availability

The raw data supporting the conclusions of this article will be made available by the authors, without undue reservation.
