# Supplementary material for: Practice behavior of first-year university music students: thriving in unusual times
Source: Front Psychol. 2026 Apr 28;17:1771837. doi: 10.3389/fpsyg.2026.1771837 (PMC13161096; doi:10.3389/fpsyg.2026.1771837)
Supplement: Supplementary file 1 [file Data_Sheet_1.pdf]

Supplementary Material

Practice behavior of first-year university music students: Thriving in unusual times

1 Complete Table 3

Statistical analyses (p-values and p.eta² in italic)	Survey (2018-2019)			Survey (2019-2020)		
	Time (T1-T2)	*Institute*	*Inter-action*	*Time (T1-T2)*	*Institute*	*Inter-action*
Daily practice time	.880<br>*.001*	.004<br>*.128*	,096<br>*.046*	.526<br>*.009*	.001<br>*.216*	.012<br>*.136*
**During my practice, I use the following strategies:**						
Play/Sing through without break	.268<br>*.020*	.007<br>*.110*	.616<br>*.004*	.025<br>*.111*	.142<br>*.048*	.483<br>*.011*
Repeat until problem is solved	.118<br>*.039*	.098<br>*.047*	.316<br>*.016*	.022<br>*.112*	<.001<br>*.255*	.585<br>*.007*
Stop when there is a problem and start over	.518<br>*.007*	.173<br>*.030*	.238<br>*.022*	.144<br>*.049*	<.001<br>*.266*	.145<br>*.049*
Identify problem and work on it	.769<br>*.001*	.854<br>*.001*	.082<br>*.044*	.733<br>*.002*	.785<br>*.001*	.104<br>*.040*
Start slowly and Increase tempo	.324<br>*.015*	.356<br>*.014*	.964<br>*.001*	.240<br>*.031*	.240<br>*.031*	.088<br>*.046*
Transpose the piece or sections of the piece	.701<br>*.002*	.434<br>*.010*	.191<br>*.021*	.832<br>*.001*	.174<br>*.042*	.141<br>*.046*
Modify the rhythm of the piece or a section	.077<br>*.049*	.634<br>*.004*	.485<br>*.008*	.611<br>*.006*	.564<br>*.008*	.381<br>*.017*
**When practicing, I use methods that I learned from:**						
Current teacher	.732<br>*.002*	.207<br>*.025*	.733<br>*.002*	.142<br>*.049*	.153<br>*.048*	.637<br>*.005*
Former teacher	.313<br>*.016*	.079<br>*.048*	.146<br>*.033*	.084<br>*.047*	.207<br>*.037*	.084<br>*.047*
Master classes	.341<br>*.014*	.344<br>*.014*	.610<br>*.004*	.850<br>*.001*	.168<br>*.045*	.850<br>*.001*
Ensemble conductor	.603<br>*.004*	.790<br>*.001*	.265<br>*.020*	.171<br>*.043*	.277<br>*.027*	.171<br>*.043*

Peers	.017<br>*.087*	.222<br>*.024*	.311<br>*.016*	.934<br>*.001*	.007<br>*.157*	.084<br>*.047*
Video tutorials	.060<br>*.049*	.037<br>*.067*	.060<br>*.049*	.809<br>*.001*	.023<br>*.114*	.405<br>*.016*
**I spent my practice time in the following manner:**						
General technique	.977<br>*.001*	.876<br>*.001*	.534<br>*.006*	.303<br>*.025*	.696<br>*.004*	.741<br>*.003*
Playing/sing through repertoire	.820<br>*.001*	.901<br>*.001*	.820<br>*.001*	.311<br>*.024*	.999<br>*.001*	.563<br>*.001*
Experimental interpretations	.702<br>*.002*	.499<br>*.007*	.707<br>*.002*	.035<br>*.099*	.964<br>*.001*	.742<br>*.003*
Improving intonation	.974<br>*.001*	.696<br>*.002*	.599<br>*.004*	.999<br>*.001*	.471<br>*.012*	.999<br>*.001*
Performing (for friends or recording)	.800<br>*.001*	.300<br>*.017*	.800<br>*.001*	.240<br>*.032*	.463<br>*.013*	.416<br>*.015*

2 Complete Table 4

**Statistical analyses (p-values and p.eta² in italic)**	**UMGer**			**UCLA**		
	Time (T1-T2)	*Survey (M1-M2)*	*Inter-action*	*Time (M1-M2)*	*Survey (M1-M2)*	*Inter-action*
Daily practice time	.029<br>*.074*	.304<br>*.017*	.593<br>*.005*	.022<br>*.119*	.498<br>*.011*	.219<br>*.036*
**During my practice, I use the following strategies:**						
Play/Sing through without break	.126<br>*.037*	.638<br>*.004*	.754<br>*.002*	.004<br>*.174*	.596<br>*.006*	.024<br>*.110*
Repeat until problem is solved	.012<br>*.097*	.235<br>*.023*	.069<br>*.049*	.105<br>*.048*	.003<br>*.189*	.958<br>*.001*
Stop when there is a problem and start over	.019<br>*.086*	.003<br>*.132*	.067<br>*.049*	.471<br>*.012*	<.001<br>*.395*	.471<br>*.012*
Identify problem and work on it	.207<br>*.026*	.029<br>*.075*	.207<br>*.026*	.310<br>*.023*	.236<br>*.031*	.310<br>*.023*
Start slowly and Increase tempo	.324<br>*.016*	.373<br>*.013*	.951<br>*.001*	.227<br>*.032*	.227<br>*.032*	.088<br>*.043*
Transpose the piece or sections of the piece	,164<br>*.032*	.012<br>*.098*	.716<br>*.002*	.039<br>*.093*	.910<br>*.001*	.210<br>*.037*
Modify the rhythm of the piece or a section	.201<br>*.026*	.338<br>*.015*	.418<br>*.011*	.673<br>*.004*	.859<br>*.001*	.190<br>*.038*
**When practicing, I use methods that I learned from:**						
Former teacher	.732<br>*.002*	.062<br>*.049*	.732<br>*.002*	.820<br>*.001*	.086<br>*.046*	.078<br>*.048*

Current teacher	.282<br>*.019*	.208<br>*.026*	.282<br>*.019*	.819<br>*.001*	.379<br>*.018*	.511<br>*.010*
Master classes	.964<br>*.001*	.460<br>*.009*	.642<br>*.004*	.585<br>*.007*	.224<br>*.033*	.585<br>*.007*
Ensemble conductor	.014<br>*.096*	.399<br>*.012*	.242<br>*.022*	.815<br>*.001*	.151<br>*.045*	.815<br>*.001*
Peers	.007<br>*.114*	.148<br>*.034*	.667<br>*.003*	.588<br>*.007*	.640<br>*.005*	.094<br>*.046*
Video tutorials	.317<br>*.016*	.028<br>*.076*	.070<br>*.049*	.431<br>*.014*	.029<br>*.102*	.431<br>*.014*
**I spent my practice time in the following manner:**						
General technique	.956<br>*.001*	.611<br>*.004*	.486<br>*.008*	.395<br>*.016*	.383<br>*.017*	.767<br>*.002*
Playing/sing through repertoire	.964<br>*.001*	.245<br>*.022*	.570<br>*.005*	.496<br>*.010*	.241<br>*.030*	.496<br>*.010*
Experimental interpretations	.172<br>*.030*	.841<br>*.001*	.580<br>*.005*	.288<br>*.025*	.535<br>*.009*	.288<br>*.025*
Improving intonation	.785<br>*.001*	.471<br>*.009*	.785<br>*.001*	.830<br>*.001*	.297<br>*.025*	.830<br>*.001*
Performing (for friends or recording)	.797<br>*.001*	.789<br>*.001*	.797<br>*.001*	.234<br>*.032*	.163<br>*.044*	.405<br>*.016*

3 Levene-Test statistics for homogeneity of variance

Based on the mean value and the tested groups were the institutions.

**Levene-Test (p-values)**	***Survey (2018-2019)***		***Survey (2019-2020)***	
	T1	T2	T1	T2
Daily practice time	.014	.119	.079	<.001
**During my practice, I use the following strategies:**				
Play/Sing through without break	<.001	<.001	.052	<.001
Repeat until problem is solved	.025	.021	.023	.009
Stop when there is a problem and start over	.509	.001	<.001	<.001
Identify problem and work on it	.208	.007	<.001	.235
Start slowly and Increase tempo	.007	.288	<.001	.029
Transpose the piece or sections of the piece	.001	.858	.103	<.001
Modify the rhythm of the piece or a section	.046	.768	<.001	.083
**When practicing, I use methods that I learned from:**				
Current teacher	.231	.106	<.001	.001
Former teacher	<.001	.152	.984	<.001

Master classes	.091	.182	.887	.100
Ensemble conductor	.006	.463	<.001	.732
Peers	.091	.275	.002	.781
Video tutorials	<.001	.029	<.001	.006
**I spent my practice time in the following manner:**				
General technique	.706	.437	.768	.235
Playing/sing through repertoire	.160	.878	.010	.404
Experimental interpretations	.165	.068	.130	.527
Improving intonation	.759	.903	.249	.336
Performing (for friends or recording)	.151	.614	.655	.218
